# Supplementary material for: Edge based metric dimension of various coffee compounds
Source: PLoS One. 2024 Apr 11;19(4):e0294932. doi: 10.1371/journal.pone.0294932 (PMC11008893; doi:10.1371/journal.pone.0294932)
Supplement: Attachment — Submitted filename: Response to reviewers.docx [file pone.0294932.s001.docx]

**Original Manuscript ID:** PONE-D-23-20879

**Original Article Title: “Edge Based Metric Dimension of Various Coffee Compounds**”

**To:** PLOS ONE

**Re:** Response to reviewers

Dear Editor,

Thank you for allowing a resubmission of our manuscript, with an opportunity to address the reviewers’ comments.

We are uploading (a) our point-by-point response to the comments (below) (response to reviewers), (b) an updated manuscript with yellow highlighting indicating changes, and (c) a clean updated manuscript without highlights (PDF main document).

Best regards,

Muhammad Azeem

**First of all, the authors are grateful to the anonymous referees for their valuable comments and suggestions that improved this paper.**

**Answer to Reviewer# 1**

Coffee includes phenolic acids, diterpenes, and caffeine in addition to being a significant dietary supply of physiologically active substances. Some coffee secondary metabolites may favourably alter a variety of anti-cancer defence mechanisms, claims a particular study. In order to comprehend the mechanics of coffee molecules, this research examined a few coffees chemical structures in terms of edge locating numbers or edge metric size. The graph theoretical features of coffee chemical structures are also included in this study. These papers pay particular attention to the compounds present in coffee, including caffeine, diterpene, kahweol, chlorogenic, caffeic, gallotannins, and ellagitannins. The paper is well written, interested and the results are good, I would like to suggest the following MINOR corrections before acceptance:

**Concern 1:** A professional proofreading revision is strongly required. Typos must be corrected.

**Response 1:** We have revised the work according to the suggestion.

**Concern 2:** Please add more details about the studied problem

**Response 2:** We have added the detail of studied accordingly.

**Concern 3:** The introduction must be reformulated to contain literature and future works, the main aim of the work. Also, the arrangement of the manuscript should be added in a paragraph at the end of the introduction.

**Response 3:** We have addressed this point and tried to make the introduction in alignment.

**Concern 4:** The authors should state clearly in the introduction the advantages of the used technique and a summary of the literature.

**Response 4:** We have added a complete section to make this point clear.

**Concern 5:** The results are interesting, also there was a great effort done, but there are NO clear applications of these results. If the authors can add some applications of their results this will be great.

**Response 5:** We have added a complete section to make this point clear.

**Concern 6:** I didn’t see any mathematical model for the problem under study, I think if there is a mathematical model that describes the studied problem it should be added or minimum referring to it in some references.

**Response 6:** No, there is no mathematical study other than proof which are already added in this work.

**Concern 7:** Some new works on the studied problem should be added, this will improve the paper.

**Response 7:** Yes, we have added new work in the revised version of this article.

**Concern 8:** The authors should revise and carefully arrange the references according to the guidelines of the journal.

**Response 8:** Yes, we have added new work in the revised version of this article.

**Answer to Reviewer # 2**

I found the article presents interesting ideas for studding the molecules found within coffee, but I have some major issues with article which prevents it from being published.

**Concern # 01:** The biggest issue is that the article does not motivate the reason why determining the edge dimension of coffee is useful. It is not clear from the article what we gain by knowing the edge dimension of coffee. I would like to see a section which explains the usefulness of edge dimension when it comes to analysing chemical compounds, to at least prime the reader into understanding what these numbers mean when it comes to chemical reactions. In the same vain, there is no discussion of the results in the conclusion which is sorely missed as a discussion of any implications of the results would ground the paper and make it far more impactful.

**Response # 01:** Yes, we have added new work in the revised version of this article.

**Concern # 02:** The article was confusing to read, particularly when it came to explaining the core methodology and core concepts. The explanation of the edge metric of a graph was unclear and by extension the edge dimension as well. Definitions 1.2 and 1.3 where hard to understand and it wasn't really clear what they were defining. The notation used by the definitions was non-standard but did not heavily impact on the clarity of the definitions. Figure 1 describes the methodology but has some quirks/mistakes in it. A step is repeated twice in Figure 1, specifically "Computing the edge resolving the graph," was this step repeated twice? Why was it repeated twice? I am guessing that it is just one step. The authors should make this clearer. In the step "Computing the edge metric dimension using Definition 1.1" Definition 1.1 is used to calculate the edge metric dimension, but Definition 1.1 does not define the edge metric dimension but only defines the edge metric. Presumably the authors meant Definition 1.3.

**Response # 02:** Thank you so much for your suggestions and very useful comments. We have addressed all the comments and make it readable.
